# Pan-cancer analysis identifies FAM49B as an immune-related prognostic maker for hepatocellular carcinoma

**DOI:** 10.7150/jca.65421

**Published:** 2022-01-01

**Authors:** Feng Xu, Jionghuang Chen, Dihua Huang

**Affiliations:** 1 Department of General Surgery, Shaoxing People's Hospital (Shaoxing Hospital, Zhejiang University School of Medicine), Shaoxing, Zhejiang, China.; 2 Department of General Surgery, Sir Run Run Shaw Hospital, Zhejiang University School of Medicine, Hangzhou, China.; 3 Department of Endocrinology, Shaoxing People's Hospital (Shaoxing hospital, Zhejiang University School of Medicine), Shaoxing, Zhejiang, China.

**Keywords:** FAM49B, TCGA, hepatocellular carcinoma, immune microenvironment, prognostic biomarker

## Abstract

Family with sequence similarity 49, member B (FAM49B) is highly expressed in many tumors, its role in malignant tumors especially in hepatocellular carcinoma (HCC) remains uncertain. We first evaluated the expression, clinical features, and prognostic value of FAM49B using RNA-seq and clinical data from The Cancer Genome Atlas. We further assessed the role of FAM49B in the tumor immune microenvironment. The correlation of FAM49B with the sensitivity of 192 anti-cancer drugs was analyzed using data from Genomics of Drug Sensitivity in Cancer database. qRT-PCR assay was used to validate the expression of FAM49B in HCC. FAM49B was expressed at high levels in most tumor types, including HCC. High FAM49B expression predicted poor survival in patients with HCC. We also found that FAM49B expression was negatively associated with the infiltration levels of immune killer cells, including NK cells, and positively associated with immunosuppressive cells, including Tregs and Central Memory T cell (Tcm), in HCC. In addition, FAM49B expression was positively associated with immune checkpoints, immune regulation genes, MHC genes, chemokines and chemokine receptors. Patients with evaluated expression of FAM49B might be resistant to several anti-cancer drugs. Our results suggest that FAM49B is a potential prognostic biomarker for HCC. FAM49B play a potential key role in regulating tumor immune microenvironment and anti-tumor drug tolerance.

## Introduction

In recent years, significant progress has been made in cancer-related treatment technologies in hepatocellular carcinoma (HCC). However, the survival rate for HCC is still very low 1,2. Studies have shown that there is a certain relationship between tumor immune microenvironment and tumor progression in HCC 3-5. The immunosuppressive microenvironment has a poor effect on the prognosis of patients with HCC. Thus, it is urgent for ameliorating the prognosis of HCC patients to detect immunosuppressive status-related prognostic marker and potential drug targets.

Family with sequence similarity 49, member B (FAM49B), localized on chromosome 8q24, encodes for a 37-kDa protein, was previously reported to involve progression of several diseases, including tumor progression 6. For example, FAM49B was up-regulated and promoted proliferation and metastasis of gallbladder cancer cell 7.

Suppressing the immune checkpoint and immunosuppressive cells, activating T cells and NK cells, provides a new idea for tumor immunotherapy 8. Interestingly, researches have reported that FAM49B could inhibit T cell activation by inhibiting Rac activity and regulating cytoskeleton reorganization 9. These reports indicate that FAM49B may play an essential role in tumor progression. However, the role of FAM49B in HCC remains uncertain.

In our study, we comprehensively analyzed the role of FAM49B in HCC and pan-cancer, including the expression, prognostic value, DNA methylation, and copy number alteration (CNA) alteration of FAM49B. The correlation between FAM49B expression and infiltration level of immune cell and immune regulation-related genes were further evaluated. The sensitiveness of anti-cancer drugs was assessed for patients with high FAM49B expression. Our results provide novel insights into the functional role of FAM49B in HCC, highlighting a potential mechanism whereby FAM49B influences the tumor immune microenvironment (TIME), as well as prognosis of tumor patients.

## Materials and methods

### Data collection

The RNA-seq and clinical data of TCGA, CCLE, and GTEx were downloaded from UCSC XENA website (https://xenabrowser.net/datapages/). The detailed criteria for the inclusion and exclusion of patients enrolled into the current study can be found in the website of TCGA (https://www.cancer.gov/about-nci/organization/ccg/research/structural-genomics/tcga/studied-cancers). Surgical resection of biopsy biospecimens were collected from patients diagnosed with hepatocellular carcinoma (HCC), and had not received prior treatment for their disease (ablation, chemotherapy, or radiotherapy). As a part of The Cancer Genome Atlas (TCGA) network, the detailed clinic parameters of enrolled patients with hepatocellular carcinoma were originally published by The Cancer Genome Atlas Research Network 10.

The gene mutation data were obtained from UCSC XENA website. The RNA-seq data were batched and normalized into log_2_(tpm+0.001). The methylation and copy number of FAM49B were downloaded from cBioPortal database (https://www.cbioportal.org/).

### Prognostic analysis

Univariate Cox regression (uniCox) and Kaplan-Meier analyses were conducted to explore the influence of FAM49B on the survival of patients in pan-cancer using R package “survminer” and “survival”. Overall survival (OS), disease-specific survival (DSS), disease-free interval (DFI), and progression-free interval (PFI) of tumor patients were evaluated.

### Gene ontology (GO), Kyoto encyclopaedia of genes and genome (KEGG), and Gene set variation analysis (GSVA)

The top 300 genes most positively associated with FAM49B were selected for enrichment analysis to reflect the function of FAM49B. GO analysis was performed using EnrichGO function in the “clusterProfiler” R package, KEGG analysis was performed using the EnrichKEGG function of the “clusterProfiler” R package. Gene Set Variation Analysis (GSVA) was conducted using the R package “GSVA” to calculate the pathway score of each sample based on the MSigDB database v7.1 (https://www.gsea-msigdb.org/gsea/msigdb/index.jsp).

### Gene set enrichment analysis (GSEA)

The correlation between FAM49B and all protein-coding mRNAs was analyzed in each tumor from TCGA cohort. The mRNAs correlated with FAM49B (Pearson's correlation coefficient, *p* < 0.05) were ranked and subjected to GSEA using R package “clusterProfiler”.

### Tumor immune microenvironment (TIME) analysis

The R package “ESTIMATE” was used to assess the stromal and immune score of each patient in TCGA cohort. We downloaded the infiltration data of 24 immune cells of 33 tumors from ImmuCellAI database (http://bioinfo.life.hust.edu.cn/ImmuCellAI#!/) and conducted the correlation analysis with FAM49B.

### Drug resistant analysis

The IC50, gene expression data, clinical information of 192 drugs and 809 cell lines were downloaded from GDSC (https://www.cancerrxgene.org/). The association between FAM49B expression and IC50 values of 192 drugs were analyzed and Spearman's correlation coefficients were calculated.

### Human tissue samples

10 pairs of HCC tissues and adjacent normal tissues were obtained from HCC patients from 2013 to 2015. The research protocols were approved by the Research Ethics Committee of Sir Run Run Shaw Hospital, School of Medicine, Zhejiang University. All participants gave written consent of their tissue samples and medical information to be analyzed for scientific research.

### qRT-PCR

Total RNA of HCC tissues was isolated and purified by miRNeasy Mini Kit (Qiagen, Maryland, USA). Quantitative PCR (qPCR) analysis of samples was performed using PrimeScript RT Reagent Kit (Takara, Otsu, Japan) following the manufacturer's instructions. ACTB was used as the control gene. The target genes and primers were designed as follows: FAM49B forward 5'-CATGCACAGACCTTGAGCAG-3' and reverse 5'-ATTGCCTCTCGTATTTCGTGG-3', ACTB forward 5′-CATGTACGTTGCTATCCAGGC-3′ and reverse 5′-CTCCTTAATGTCACGCACGAT-3′.

## Results

### Pan-cancer expression of FAM49B

**Figure 1:**
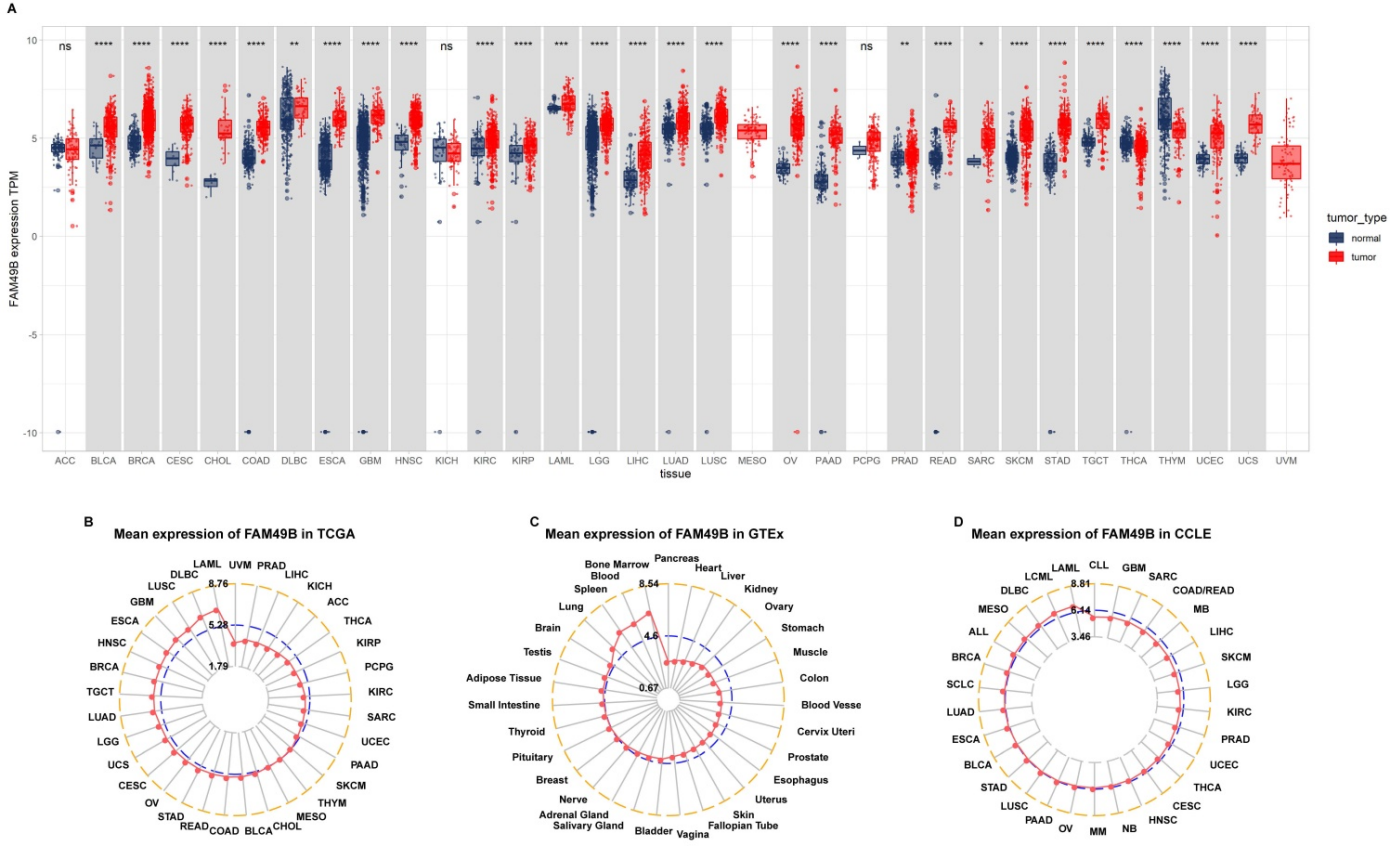
** Expression of FAM49B. (A)** pan-cancer expression of FAM49B. **(B)** FAM49B expression in tumor tissues from TCGA cohort. **(C)** FAM49B expression in normal tissues from GTEx cohort. **(D)** FAM49B expression in cancer cell lines from CCLE cohort. *p < 0.05, **p < 0.01, ***p < 0.001, ****p < 0.0001.

We first assessed the expression of FAM49B in 31 tumor types with both tumor and normal tissues using TCGA and GTEx data. Results revealed that FAM49B was over-expressed in 26 of 31 tumors, including BLCA, BRCA, CESC, CHOL, COAD, DLBC, ESCA, GBM, HNSC, KIRC, KIRP, LAML, LGG, LIHC, LUAD, LUSC, OV, PAAD, PRAD, READ, SARC, SKCM, STAD, TGCT, UCEC and UCS, while only lowly expressed in THCA and THYM (Figure 1A). By evaluating the FAM49B expression only in tumor tissues, we observed that FAM49B was highest expressed in LAML and lowest in UVM (Figure 1B). In normal tissues from the GTEx database, results revealed that FAM49B expression was highest in bone marrow and lowest in pancreas (Figure 1C). As for tumor cell lines, we found that FAM49B expression was highest in LAML cell lines using data from the CCLE database (Figure 1D).

**Figure 2:**
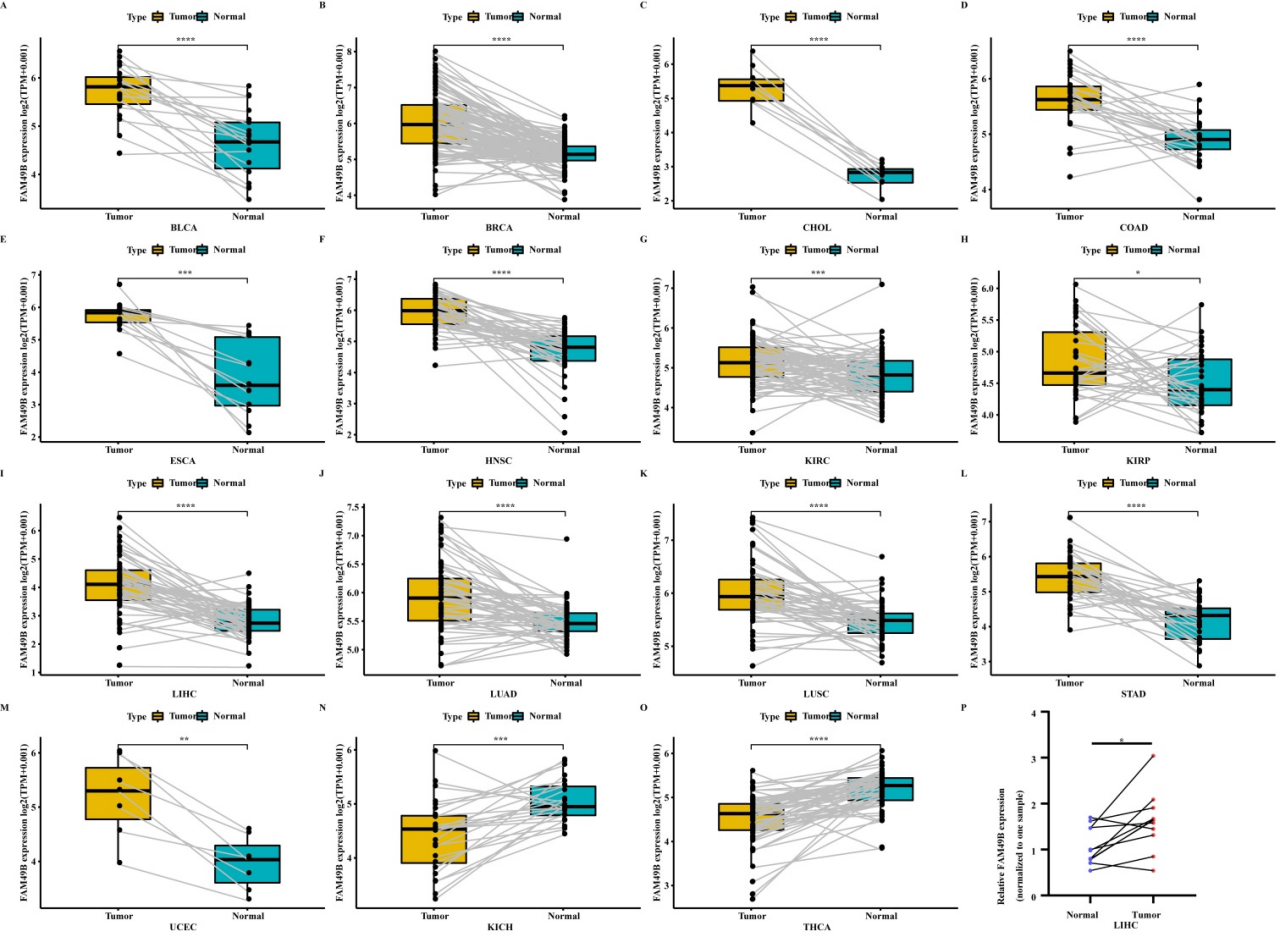
** Expression of FAM49B in paired tumor and adjacent normal tissues. (A-O)** FAM49B expression in paired tumor and adjacent normal tissues from TCGA in indicated tumor types.** (P)** qRT-PCR assay evaluates the FAM49B expression in paired tumor and adjacent normal tissues from HCC patients. *p < 0.05, **p < 0.01, ***p < 0.001, ****p < 0.0001.

**Figure 3:**
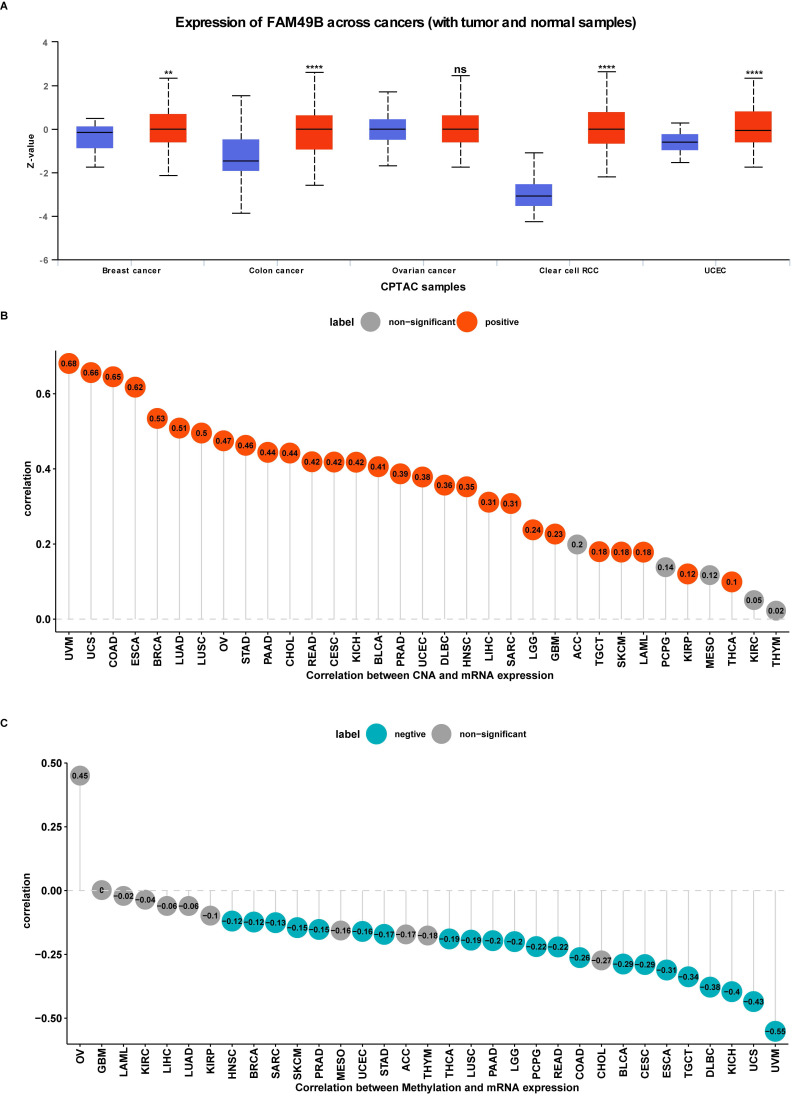
** Protein level, CNA and methylation of FAM49B. (A)** The protein level of FAM49B in indicated tumor types using Ualcan database. **(B)** The correlation between FAM49B expression and CNA. **(C)** The correlation between FAM49B expression and DNA methylation.

We further evaluated the FAM49B expression in paired tumor and adjacent normal tissues. FAM49B was over-expressed in tumor tissues in BLCA, BRCA, CHOL, COAD, ESCA, HNSC, KIRC, KIRP, LIHC, LUAD, LUSC, STAD, and UCEC (Figure 2A-M). In contrast, FAM49B was lowly expressed in tumor tissues of KICH and THCA (Figure 2N-O). The expression analysis of FAM49B in LIHC based on tumor grade was performed (Supplementary Figure 1). The results showed that the expression of FAM49B was positively correlates with the tumor grade in LIHC. In addition, qRT-PCR assay confirmed that FAM49B expression was higher in HCC tissues compared to adjacent normal tissues (Figure 2P). Furthermore, the protein level of FAM49B was higher in BRCA, COAD, KIRC, and UCEC using Ualcan database (Figure 3A).

### Gene alteration of FAM49B

We further evaluated the CNA and methylation status of FAM49B in pan-cancer. For the association between FAM49B and CNA, we found that FAM49B expression were positively correlated with CNA in LIHC (r = 0.31, *p <* 0.05) (Figure 3B). We further proved that the promoter methylation level of FAM49B have little relationship with FAM49B expression (r = -0.06, *p* > 0.05) in LIHC (Figure 3C). These results indicated that the CNA status mainly contribute to the high expression of FAM49B.

### Prognostic significance of FAM49B

**Figure 4:**
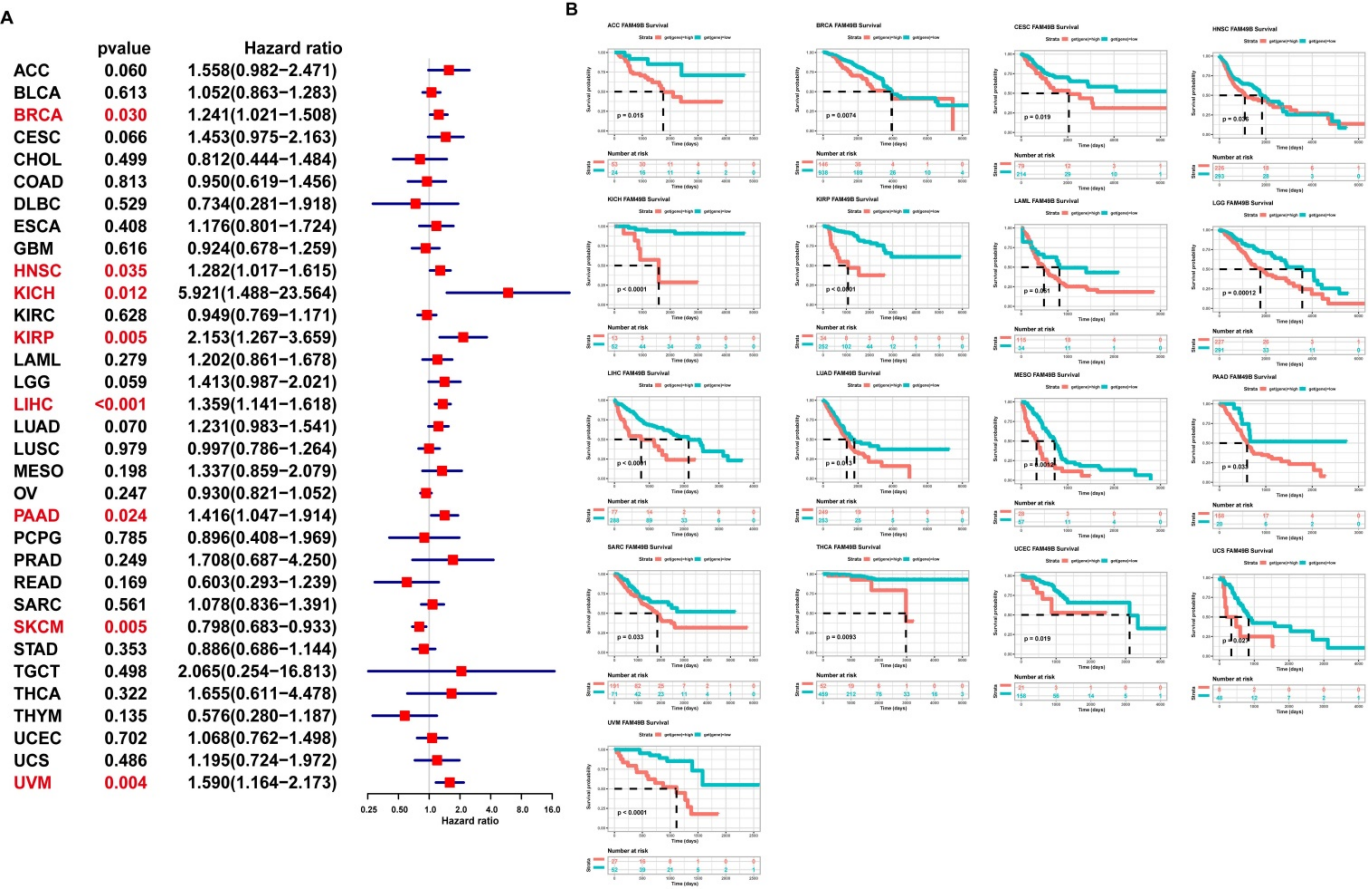
** Prognostic significance of FAM49B for OS of patients. (A)** The uniCox results of FAM49B in pan-cancer OS of patients. Red colors represent significant results (*p <* 0.05). **(B)** Kaplan-Meier OS results of FAM49B in pan-cancer. The best cut-off of FAM49B expression was set as cut-off value.

To evaluate the prognostic role of FAM49B in HCC and pan-cancer, we first conducted uniCox analysis. The uniCox overall survival results indicated that FAM49B was a risk factor in BRCA, HNSC, KICH, KIRP, LIHC, PAAD, and UVM and protective factor only in SKCM (Figure 4A). For the Kaplan-Meier analysis of OS, we observed that evaluated FAM49B expression predicted worse OS of patients in 17 tumors in TCGA cohort, such as ACC, BRCA, CESC, HNSC, KICH, KIRP, LAML, LGG, LIHC, LUAD, MESO, PAAD, SARC, THCA, UCEC, UCS, and UVM (Figure 4B).

**Figure 5:**
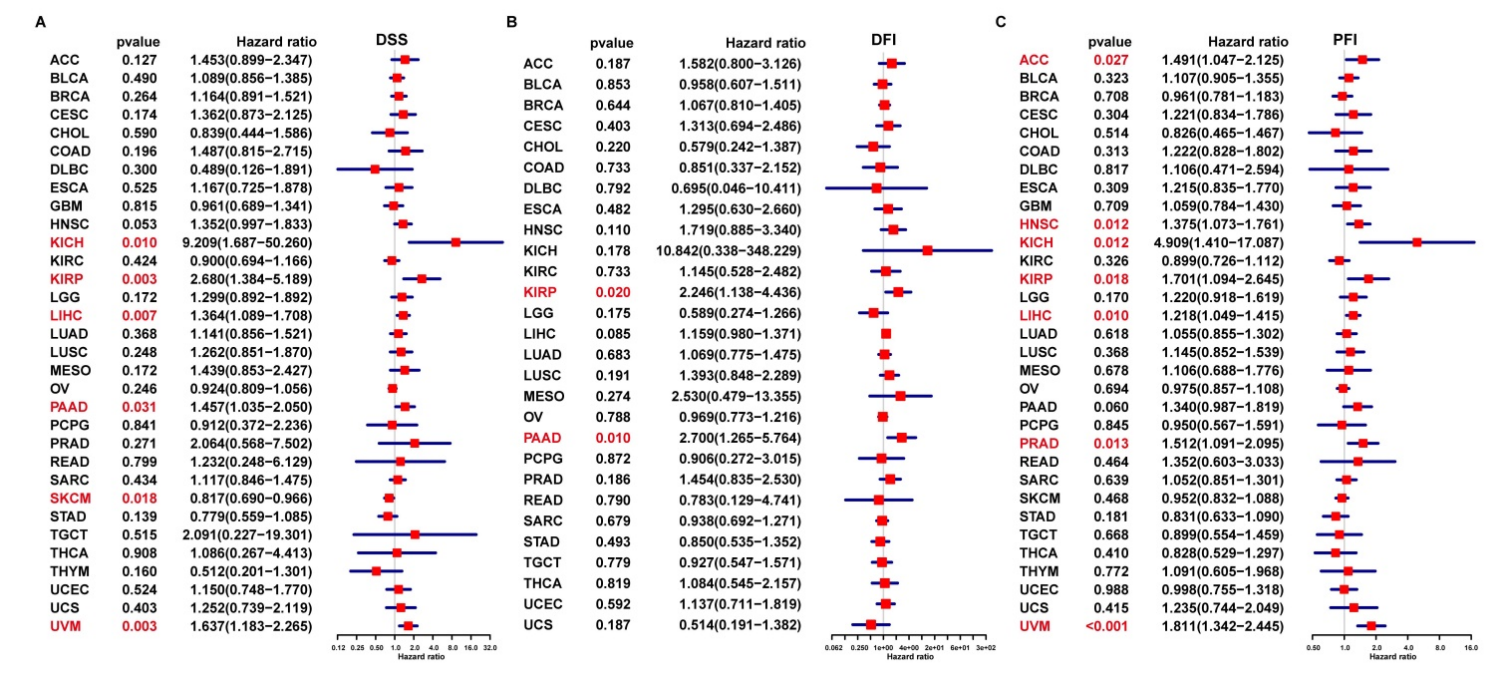
** Prognostic significance of FAM49B for DSS, DFI, and PFI of patients. (A-C)** The uniCox results of FAM49B in pan-cancer. For DSS (disease-specific survival) **(A)**, DFI (disease-free interval) **(B)**, and PFI (progression-free interval) **(C)** of patients. Red color represents significant results (*p <* 0.05).

In addition, the uniCox DSS results indicated that FAM49B was a risk factor in KICH, KIRP, LIHC, PAAD, and UVM (Figure 5A). For DFI results, FAM49B was a risk factor in KIRP and PAAD (Figure 5B). For PFI, a high FAM49B expression predicted shorter PFI times in patients with ACC, HNSC, KICH, KIRP, LIHC, PRAD, and UVM (Figure 5C). These results indicated that FAM49B was a prognostic marker in HCC and other tumors.

### Enrichment analyses of FAM49B

**Figure 6:**
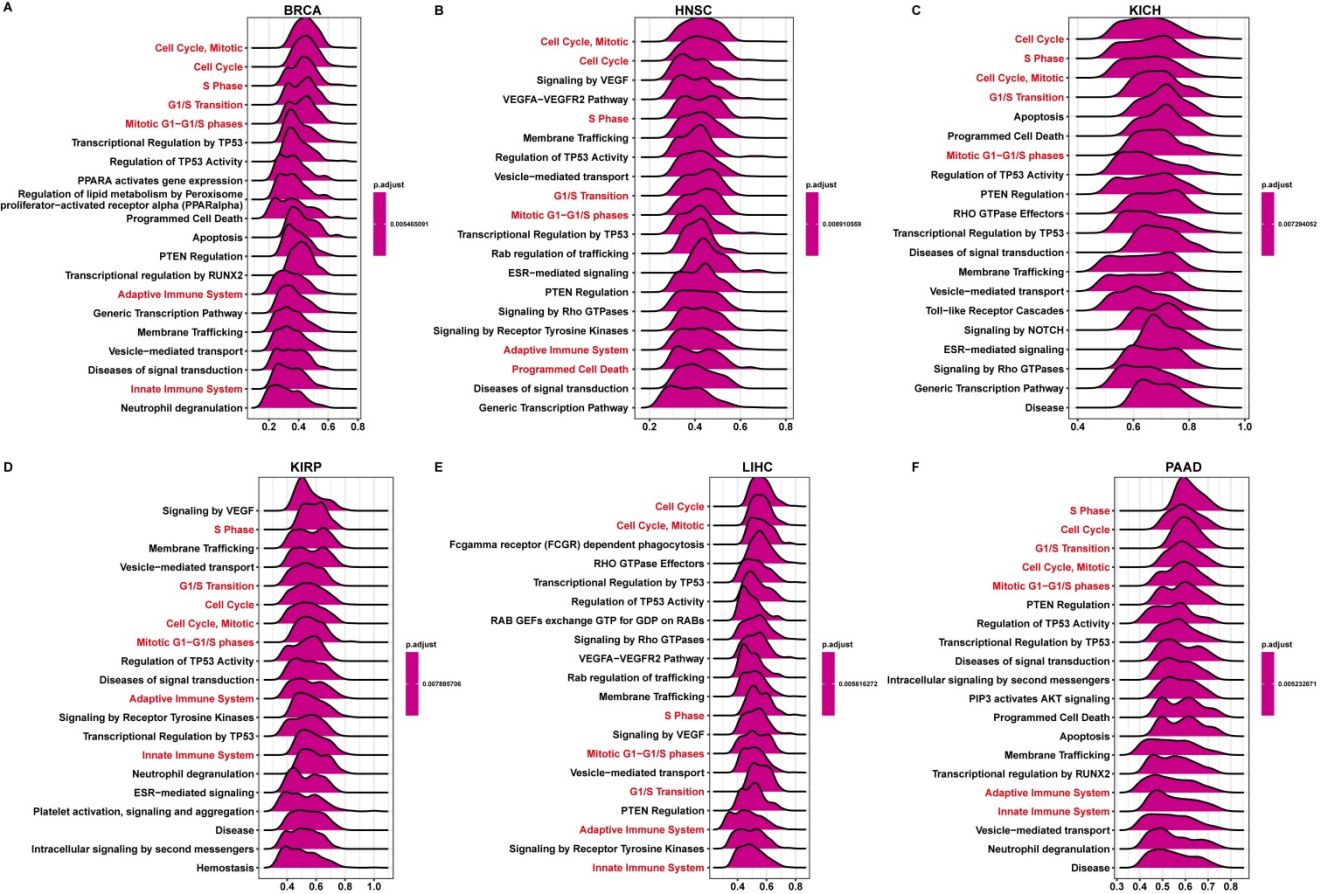
** GSEA of FAM49B. (A-F)** The top 20 GSEA-Reactome results were showed in indicated tumors.

Next, we conducted the GSEA to predict the pathways FAM49B might involve. The genes correlated with FAM49B (*p <* 0.05) were ranked and used to perform GSEA. We analyzed the reactome pathway (GSEA-Reactome) terms using R package “clusterprofiler” in pan-cancer. The results revealed that FAM49B was enriched in the cell cycle-related pathways (such as Cell Cycle and S Phase) and immune regulation-related pathways (Adaptive Immune System and Innate Immune System) in most tumor types, including BRCA, HNSC, KICH, KIRP, LIHC, and PAAD (Figure 6A-F). The top 20 results of gene ontology (GO), Kyoto encyclopaedia of genes and genome (KEGG), and Gene set variation analysis (GSVA) in FAM49B was also performed (Supplementary Figure 2).

### TIME analysis

**Figure 7:**
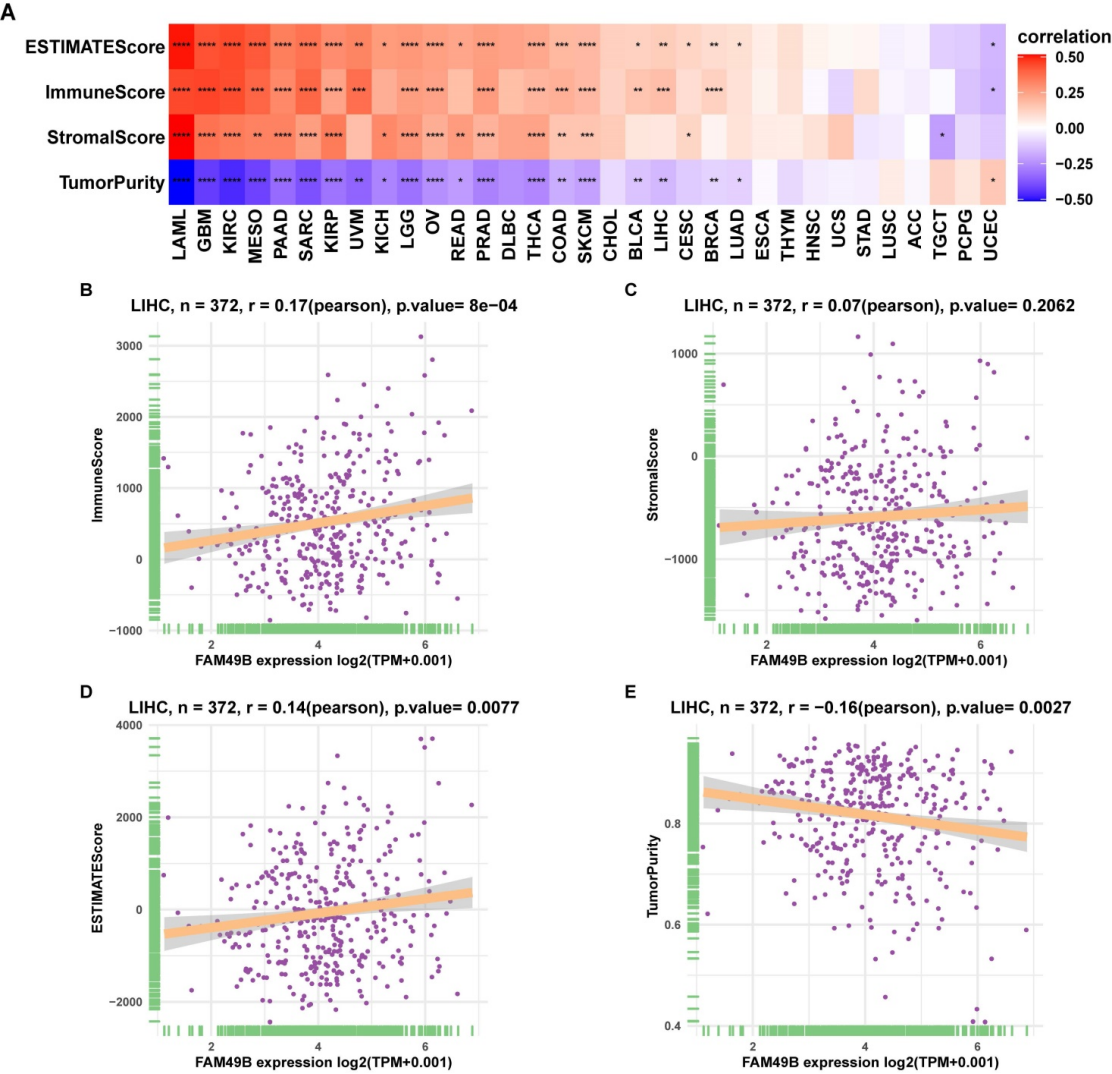
** Tumor microenvironment analysis of FAM49B. (A)** Heatmap represents the correlation between FAM49B expression and TME scores in pan-cancer. (B-E) The correlation between FAM49B expression and immune score **(B)**, stromal score **(C)**, ESTIMATE score **(D)**, and tumor purity score **(E)**. *p < 0.05, **p < 0.01, ***p < 0.001, ****p < 0.0001.

To understand the role of FAM49B in TIME of HCC, we performed the correlation analysis between FAM49B expression and stromal and immune scores calculated by R package “ESTIMATE”. Results indicated that FAM49B was positively correlated with stromal score, ESTIMATE score, and immune score and negatively correlated with tumor purity in most tumor types from TCGA (Figure 7A). While for LIHC of TCGA, FAM49B was positively associated with immune score and ESTIMATE score and negatively associated with tumor purity score, with no correlation with stromal score (Figure 7B-E).

**Figure 8:**
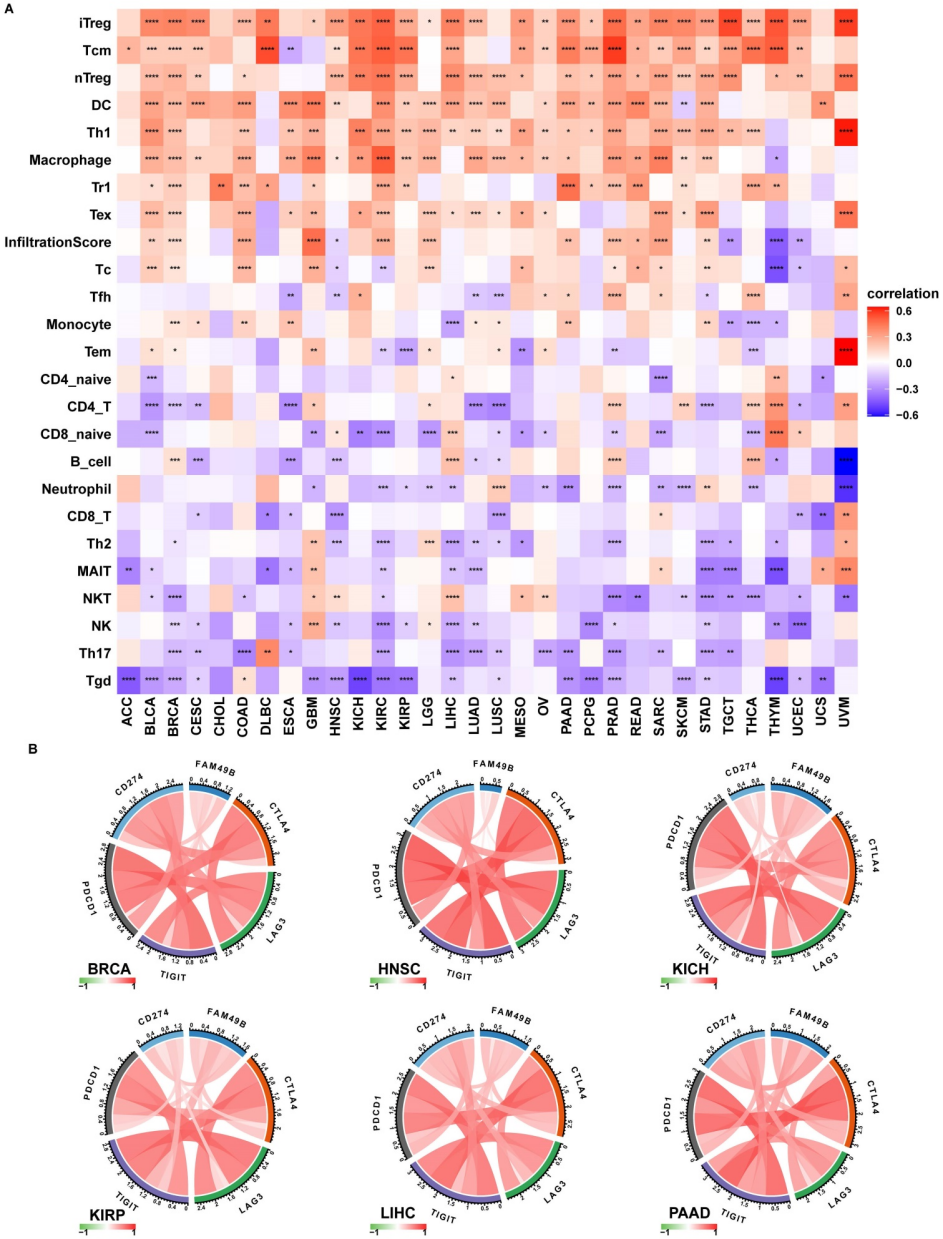
** Immune infiltration analysis. (A)** The correlation between FAM49B expression and infiltration levels of 24 immune cells. Red represents positive correlation, blue represents negative correlation, and the darker the color, the stronger the correlation. **(B)** The correlation between FAM49B expression and immune checkpoints in indicated tumors. *p < 0.05, **p < 0.01, ***p < 0.001, ****p < 0.0001.

To prove the immune-regulation function of FAM49B, we downloaded the infiltration level of 24 immune cells from the ImmuCellAI database. Results of the correlation analysis suggested that FAM49B expression was positively correlated with immunosuppressive cells, such as iTreg, Tcm, and nTreg cells, in HCC (Figure 8A).

**Figure 9:**
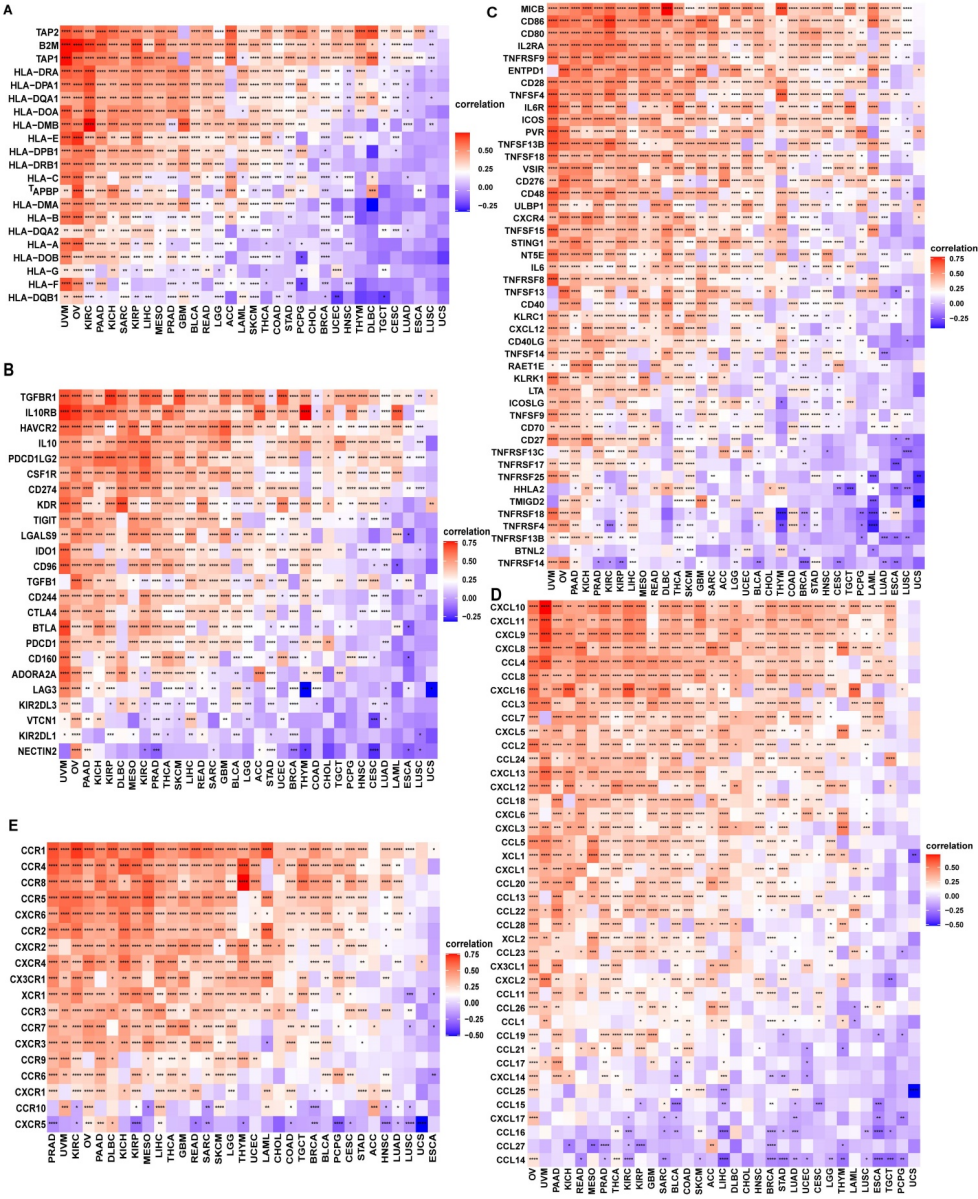
** Correlation between immunosuppressive genes. (A)** The correlation between FAM49B expression and MHC genes, immunosuppressive genes **(B)**, immune activating genes **(C)** chemokines **(D)** and chemokine receptors **(E)**. *p < 0.05, **p < 0.01, ***p < 0.001, ****p < 0.0001.

We also proved that FAM49B expression was positively correlated with immune checkpoints (CTLA4, LAG3, TIGIT, PDCD1, and CD274) in BRCA, HNSC, KICH, KIRP, LIHC, and PAAD (Figure 8B). We further explored the association between FAM49B and immune related genes. As shown in Figure 9, FAM49B was closely associated with MHC genes (Figure 9A), immunosuppressive gene (Figure 9B), immune activated genes (Figure 9C), chemokines (Figure 9D) and chemokine receptors (Figure 9E) in most tumors including HCC. These results suggested that FAM49B play an essential role in tumor immunomodulatory process.

### Drug resistance analysis

**Figure 10:**
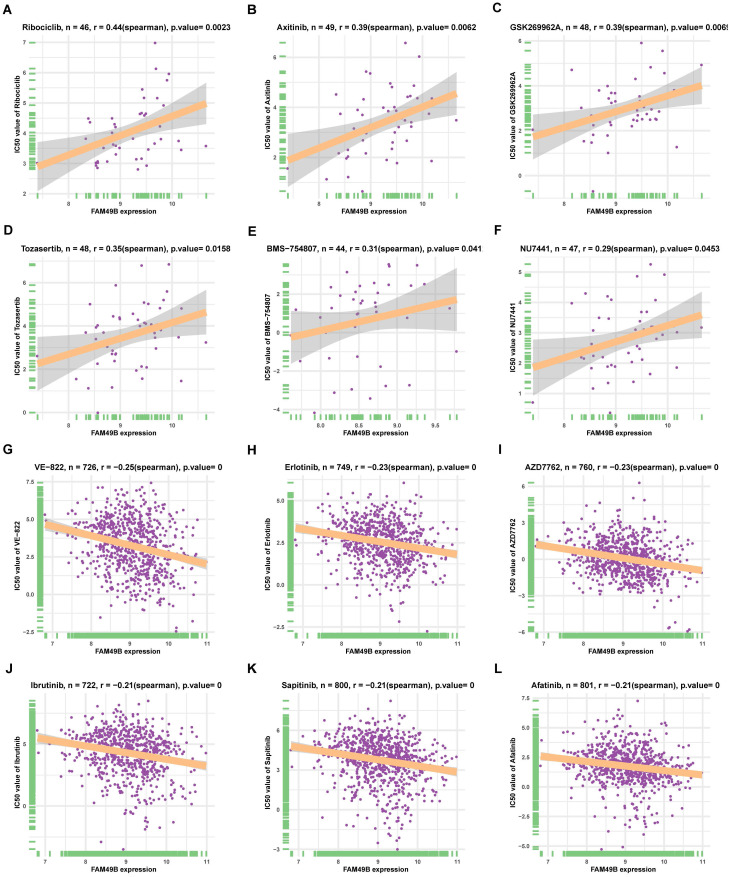
The correlation between FAM49B expression and IC50 values of anti-cancer drugs. **(A-L)** The correlation between FAM49B expression and IC50 values of indicated anti-cancer drugs.

Additionally, we analyzed the correlation between FAM49B and IC50 of 192 drugs. Among the 192 anti-cancer drugs, FAM49B expression was positively correlated with IC50 of 8 anti-cancer drugs, such as Ribociclib, Axitinib, GSK269962A, Tozasertib, BMS-754807, and NU7441 (Figure 10A-F). In contrast, FAM49B expression was negatively correlated with IC50 of 132 anti-cancer drugs, such as VE-822, Erlotinib, AZD7762, Ibrutinib, Sapitinib, and Afatinib (Figure 10G-L, Supplementary Table 1).

### Methyladenosine (m6A) related genes analysis

Methyladenosine is the most prevalent reversible methylation in mRNA and has critical roles in the tumorigenesis. The correlation analysis between FAM49B and m6A related genes was performed in pan-cancer. The data showed that FAM49B was positively correlates with m6A related genes in LIHC (Supplementary Figure 3).

## Discussion

Although some progress has been made in cancer-related treatment technology in recent years, the five-year survival rate of HCC is still very low, mainly because most patients are diagnosed in the final stage 11-13. Recent studies have shown that the remodeling of TIME by cancer cells plays an important role in the development of HCC, weakening the response of HCC patients to treatment and leading to worse survival status 14,15. Thus, the identification of essential genes that could affect TIME is urgently needed.

FAM49B was previously reported to involve in the progression of several diseases, including tumor 6. For example, FAM49B was up-regulated and promoted proliferation and metastasis of gallbladder cancer cell 7. However, its role in HCC remains unclear. In our study, we firstly assessed the expression of FAM49B and found the FAM49B expression was higher in tumor tissues compared with normal tissues in 26 of 31 tumors including HCC. In addition, qRT-PCR assay was used to validate the expression of FAM49B in HCC.

To evaluate the prognostic significance of FAM49B, we performed the uniCox and Kaplan-Meier survival analysis in TCGA cohort. Kaplan-Meier OS analysis proved that an elevated FAM49B expression predicted poorer OS of patients with HCC and other 16 tumors in TCGA cohort. The results of uniCox indicated that FAM49B was a risk factor for the OS, DSS, and PFI of HCC patients.

To explore the mechanism of FAM49B affecting prognosis, we performed the GSEA and found that the immune regulation relevant pathways were simultaneously enriched in HCC, indicating an essential role of FAM49B TIME. Thus, we further validated the results using immune cell infiltration data from the ImmuCellAI database. Results suggested that FAM49B was positively correlated with immunosuppressive cells, including iTreg, nTreg, and Tcm cells in HCC. In contrast, FAM49B was negatively correlated with immune killer cells, including NK cells in HCC. In addition, we proved that FAM49B expression was positively correlated with immune checkpoints (CTLA4, LAG3, TIGIT, PDCD1, and CD274), MHC genes, immunosuppressive genes, immune activated genes, chemokines, and chemokine receptors in most tumors including HCC. These results suggested that FAM49B play an essential role in tumor immunomodulatory process.

Additionally, we also performed the correlation analysis between FAM49B and IC50 of 192 anti-cancer drugs. We found that patients with high FAM49B expression might be resistant to several anti-cancer drugs, such as Ribociclib, Axitinib, GSK269962A, Tozasertib, BMS-754807, and NU7441.

Our findings suggest that FAM49B is a potential prognostic biomarker for HCC. FAM49B play a potential key role in regulating tumor immune microenvironment and anti-tumor drug tolerance.

## Supplementary Material

Supplementary figures.

## Abbreviations

ACC: Adrenocortical carcinoma
BLCA: Bladder Urothelial Carcinoma
BRCA: Breast invasive carcinoma
CESC: Cervical squamous cell carcinoma and endocervical adenocarcinoma
CHOL: Cholangiocarcinoma
COAD: Colon adenocarcinoma
DLBC: Lymphoid Neoplasm Diffuse Large B-cell Lymphoma
ESCA: Esophageal carcinoma
GBM: Glioblastoma multiforme
HNSC: Head and Neck squamous cell carcinoma
KICH: Kidney Chromophobe
KIRC: Kidney renal clear cell carcinoma
KIRP: Kidney renal papillary cell carcinoma
LAML: Acute Myeloid Leukemia
LGG: Lower Grade Glioma
HCC/LIHC: Liver hepatocellular carcinoma
LUAD: Lung adenocarcinoma
LUSC: Lung squamous cell carcinoma
MESO: Mesothelioma
OV: Ovarian serous cystadenocarcinoma
PAAD: Pancreatic adenocarcinoma
PCPG: Pheochromocytoma and Paraganglioma
PRAD: Prostate adenocarcinoma
READ: Rectum adenocarcinoma
SARC: Sarcoma
SKCM: Skin Cutaneous Melanoma
STAD: Stomach adenocarcinoma
TGCT: Testicular Germ Cell Tumor
THCA: Thyroid carcinoma
THYM: Thymoma
UCEC: Uterine Corpus Endometrial Carcinoma
UCS: Uterine Carcinosarcoma
UVM: Uveal Melanoma
TCGA: The Cancer Genome Atlas
CCLE: Cancer Cell Line Encyclopedia
GTEx: Genotype-Tissue Expression
GDSC: Genomics of Drug Sensitivity in Cancer database
OS: overall survival
DSS: disease-specific survival
DFI: disease-free interval
PFI: progression-free interval
