# Supplementary material for: Pan-cancer analysis identifies FAM49B as an immune-related prognostic maker for hepatocellular carcinoma
Source: J Cancer. 2022 Jan 1;13(1):278–89. doi: 10.7150/jca.65421 (PMC8692685; doi:10.7150/jca.65421)
Supplement: Supplementary file 1 — Supplementary figures. [file jcav13p0278s1.pdf]

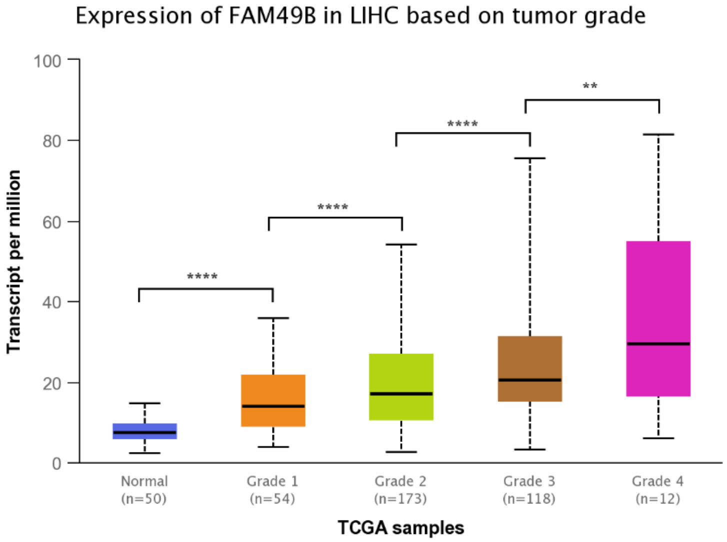

Supplementary Figure 1. The expression of FAM49B in LIHC/HCC based on tumor grade. **$p < 0.01$, ****$p < 0.0001$.

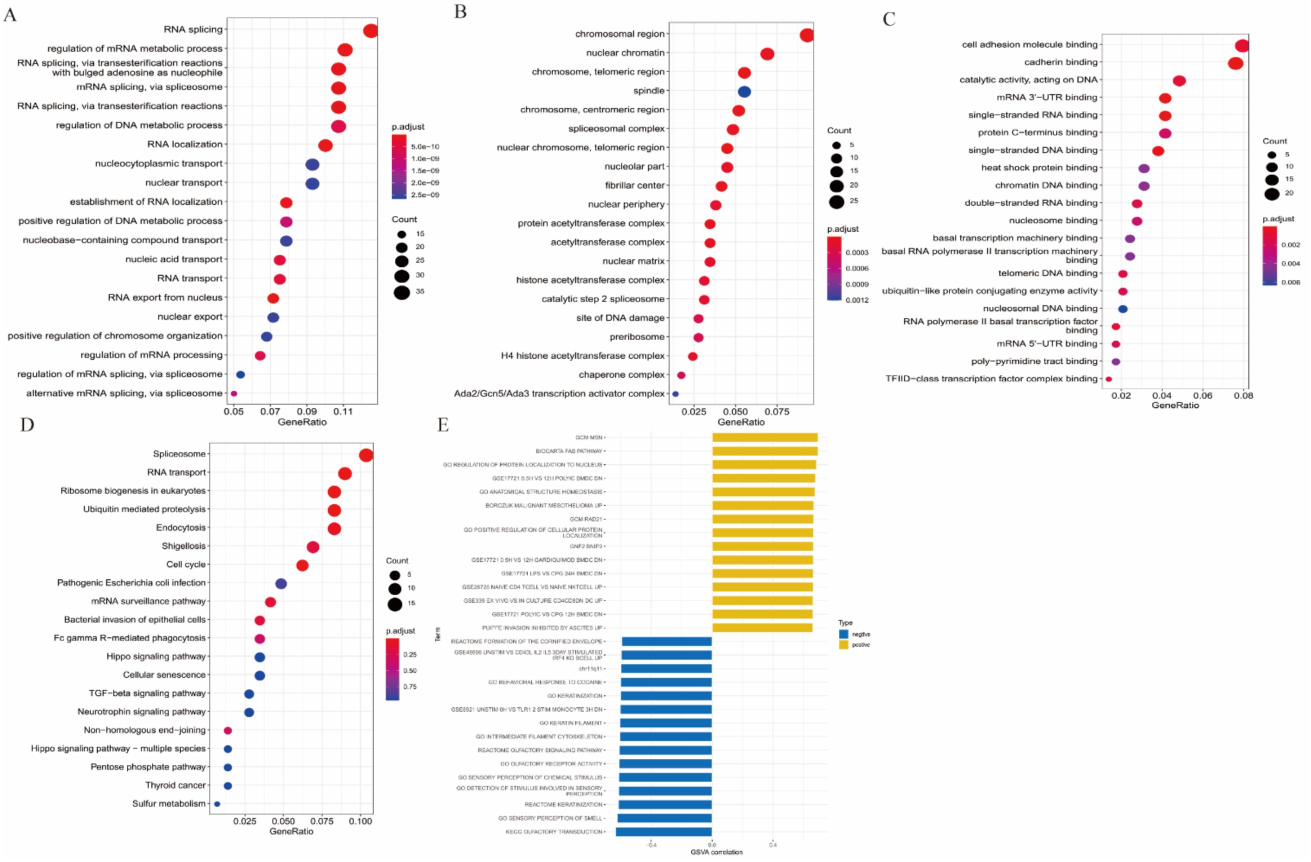

Supplementary Figure 2. The top 20 results of GO, KEGG and GSVA in FAM49B. (A) GO_BP analysis. **(B)** GO_CC analysis. **(C)** GO_MF analysis. **(D)** KEGG analysis. **(E)** GSVA.

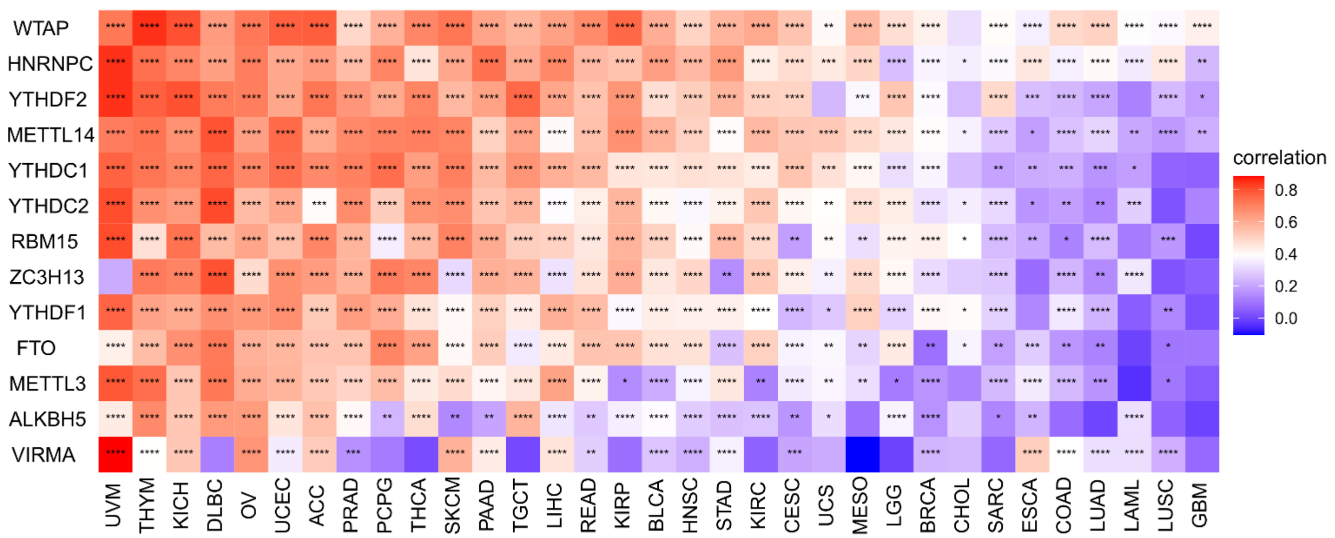

Supplementary Figure 3. The correlation analysis between FAM49B and m6A related genes.

The data showed that FAM49B was positively correlates with m6A related genes in LIHC.
